# Investigating the Efficacy of AI-Powered Innovations in ECG Analysis and Continuous Heart Monitoring: A Comprehensive Narrative Review

**DOI:** 10.7759/cureus.89743

**Published:** 2025-08-10

**Authors:** Nehal Revuri, Quang Dai La, Marc Faltas, Francis Pryor, Sumalatha Aradhya, Jasneel S Kahlam

**Affiliations:** 1 Neurological Surgery, The Innovative STEMagazine, College Station, USA; 2 Medicine, The Innovative STEMagazine, College Station, USA; 3 Osteopathic Medicine, Rowan-Virtua School of Osteopathic Medicine, Stratford, NJ, USA, Stratford, USA; 4 Medicine, Lake Erie College of Osteopathic Medicine, Erie, USA; 5 Computer Science and Engineering, Siddaganga Institute of Technology, Tumkur, IND; 6 Internal Medicine, Stony Brook Southampton, Hackettstown, USA

**Keywords:** artificial intelligence, cardiology, continuous heart monitoring, ecg analysis, medicine

## Abstract

Cardiovascular diseases (CVD) continue to be the primary global reason for death, while arrhythmias and other structural heart diseases create major diagnostic obstacles. The combination of artificial intelligence (AI) with electrocardiogram (ECG) analysis and continuous cardiac monitoring provides transformative capabilities for detecting diseases early and delivering individualized care and better patient results. The current study reviews the effectiveness, obstacles, and potential future directions of AI-based innovations in cardiovascular healthcare.

The research used a complete literature review, which extracted information from PubMed/MEDLINE and Google Scholar and Scopus databases. The selection process for studies included only English-language research that explored AI applications in cardiovascular diagnostics. The search used "AI in ECG analysis" and "predictive analytics in heart monitoring" terms to identify relevant studies, which underwent qualitative analysis for extracting essential themes and findings.

The analysis examined 11 research manuscripts that demonstrated how AI technology improves ECG interpretation by detecting subclinical patterns with high sensitivity and specificity to diagnose arrhythmias early. The combination of continuous cardiac monitoring with AI predictive analytics proved effective in detecting early clinical deterioration while minimizing diagnostic inconsistencies. The implementation of AI solutions faces ongoing challenges because of data privacy issues and device compatibility problems, and the requirement for real-world testing.

AI-based advancements in cardiovascular diagnostics show great potential to transform clinical practice through individualized, precise medical interventions. These innovations face ongoing obstacles, but they create a fundamental advancement toward better cardiovascular health management. The full realization of AI's potential for patient care transformation depends on addressing the identified limitations.

## Introduction and background

The number-one cause of death globally, responsible for 17 million annual fatalities worldwide, is cardiovascular disease (CVD), according to the World Health Organization [1]. CVD involves vascular diseases (coronary artery disease), structural heart diseases (valvular and septal abnormalities), and electrical diseases (arrhythmias), which are recognized as separate entities [2]. Structural heart diseases arise from congenital or acquired abnormalities of the cardiac walls, valves, or primary vessels [3, 4]. Coronary artery disease (CAD) is classified as a vascular disease and not a structural disease. Most heart diseases arise from a sedentary lifestyle with unhealthy diets, or genetic factors. Common additional risk factors include hypertension, smoking, diabetes, and environmental exposures. According to United States data, CAD stands as the most prevalent heart disease among Americans because people consume excessive amounts of cholesterol in their diets [5, 6]. The disease is referred to as CAD because it develops through plaque accumulation that causes atherosclerosis, or blood vessel hardening. Where CAD leads to blood vessel dysfunction, structural heart disease deals with what happens to the physical structure of the heart [7]. Not all heart diseases belong to this category since arrhythmias are classified as irregular heart rhythm conditions. An irregular heart rhythm develops when disrupted electrical signals used for heartbeat coordination [8, 9]. Medical professionals can easily miss intermittent arrhythmias for extensive periods until patients develop other noticeable symptoms, which makes these conditions difficult to detect through testing. These untreated diseases will progressively degrade heart function until they either cause cardiac arrest or heart failure. The evolution of CVD and its harmful effects on patients serves as the essential factor for understanding the value of AI technologies and their potential impact on cardiovascular healthcare. 

Since the American College of Cardiology began operations in 1949, clinical cardiology has experienced a major transformation through the integration of AI technology. The development of AI in medicine began in the 1970s with the development of expert systems, with MYCIN recommending antibiotics for bacterial infections [10]. While not directly applicable to cardiology, MYCIN illustrated the potential of AI to provide clinical support. The healthcare industry received its official impact from AI when the MYCIN program started treating severe bacterial infections through personalized antibiotic advice for doctors in 1970 [10]. The practical application of expert systems in medicine became evident after MYCIN's influential development in the early 1970s, which led to the establishment of the American Association for Artificial Intelligence (now AAAI) in 1979 to promote AI research and responsible application [11, 12]. However, detailed historical context should not detract from the present applications in cardiovascular healthcare. AI technology has produced substantial advancements in data collection and surgical accuracy and improved healthcare quality standards [13, 14]. The sequence of development established the foundation for AI advancements, including machine learning and industrial robots, and eventually led to computer systems and voice recognition algorithms [13]. While industrial robotics is an important part of the AI landscape, we mention it less and leave it out when possible for brevity and focus. These historical events laid down the essential groundwork for upcoming AI breakthroughs in cardiovascular medicine.

The AI subfield of Machine Learning (ML) discovers patterns to generate complex algorithms for performing complicated operations. The different stages of ML operate independently to perform their specific tasks efficiently. Neural networks represent a type of ML processing system that analyzes data relationships through algorithms [15]. The two main categories of neural networks exist as convolutional neural networks (CNNs) and recurrent neural networks (RNNs) to serve separate functions. The processing of image-based datasets requires CNNs, whereas RNNs perform ML operations on sequential data [16]. Multiple neural networks operate together to form the overall term known as deep learning (DL) [13]. AI is a comprehensive area of study that covers ML and DL; cognitive computing refers to the systems that are invented to simulate human thought processes, but is not a functional subfield like ML or DL. The integration of AI combines ML and DL algorithms and cognitive computing as a subfield of AI, which applies reasoning and machine learning to solve problems and enhances human decision pathways [17]. The algorithms used by AI models extract information from data patterns to generate conclusions, which will receive thorough examination throughout this review [18]. These AI terms have related meanings, yet they represent different concepts that do not replace one another. Evaluation of healthcare AI models is done using sensitivity (true positive rate), specificity (true negative rate), and accuracy (the rate of total correctness). Balanced accuracy, sensitivity, and precision are important in achieving clinical validity and minimizing diagnostic risk [19].

The development of AI technology has emerged as a transformative diagnostic tool for cardiovascular medicine through its applications in ECG analysis and continuous cardiac monitoring. AI technology enables clinicians to perform diagnostic tasks beyond human capabilities by detecting hidden patterns in large datasets that traditional methods cannot detect [20]. The ECG has evolved from its basic diagnostic role to become a powerful screening tool that detects cardiac and non-cardiac conditions in people who show no symptoms [20]. The implementation of ongoing ECG monitoring has become essential for AI-based predictive analytics because it enables the detection of early clinical decline indicators through objective and detailed data interpretation. The continuous monitoring systems track individual patient patterns while resisting practice-based biases to generate interpretable disease indicators that support clinical decisions [21].

## Review

Methods

The article provides a thorough evaluation of AI-powered innovations used for ECG analysis and continuous heart monitoring. We conducted an extensive review of literature and empirical evidence to explore outcomes, challenges, and future prospects for AI-powered innovations in the region.

We analyzed different studies and reports and articles through PubMed/MEDLINE and Google Scholar and Scopus databases using qualitative analysis to extract essential themes and findings. The inclusion criteria selected English-language sources about AI-powered cardiovascular healthcare innovations while excluding insufficient data reports and conference abstracts and opinion pieces and duplicate records and non-English sources.

The search included the terms “AI in ECG analysis,” “Artificial intelligence in cardiovascular diagnostics,” “Continuous ECG monitoring and AI,” and “Predictive analytics in heart monitoring” and examined indicators such as “medical interventions” and “financial constraints.” We examined references from recent reviews to achieve a complete evaluation. Methodological details are summarized in Table 1.

**Table 1:** Summary of methodology. AI: artificial intelligence, ECG: electrocardiogram

Methodology steps	Description
Literature search	PubMed/MEDLINE, Google Scholar, Scopus
Inclusion criteria	Full-text articles published in English.
	Studies addressing the efficacy of AI-powered innovations in cardiovascular healthcare.
Exclusion criteria	Conference abstracts, opinion pieces, and duplicate records.
	Studies lacking sufficient data or relevance to the topic.
	Non-English language studies.
Search terms	"AI in ECG analysis"
	"Artificial intelligence in cardiovascular diagnostics" - "AI-based ECG interpretation advantages"
	"AI-based ECG interpretation advantages"
	"Super-human diagnostic AI ECG"
	"Continuous ECG monitoring and AI"
	"Predictive analytics in heart monitoring"
	"AI-based real-time heart monitoring tools”
Additional search criteria	Focus on publications from the past 5 years to ensure the relevance of emerging AI technologies.
	Peer-reviewed journals (e.g., Nature Digital Medicine, Circulation, Journal of Electrocardiology).
	Studies discussing AI's diagnostic precision, scalability, and predictive capabilities in ECG or continuous heart monitoring.

Assessment of risk of bias

Following the removal of duplicates, items were assessed for relevance based on title and abstract. Full text appraisal and risk of bias were conducted by two independent reviewers (N.R. and Q.L.) on studies deemed potentially eligible; disputes were settled with the third author (M.F.). We used specific criteria (low, some concerns, high) to classify the risk of bias shown in Table 2 [22-32].

**Table 2:** Risk of Bias Assessment ➕: Low, ➖: High, ❔: Unclear risk of bias

	Martínez-Sellés and Marina-Breysse, 2023 [22]	Muzammil et al., 2024 [23]	Salerno et al., 2003 [24]	Siontis et al., 2021 [25]	Lima et al., 2021 [26]	Adedinsewo et al., 2023 [27]	Quartieri et al., 2022 [28]	Dampage et al., 2021 [29]	Anand et al., 2024 [30]	Krittanawong et al., 2021 [31]	Shumba et al., 2023 [32]
Bias arising from the randomization process	➕	➖	❔	➕	❔	➕	➖	➕	➕	❔	➕
Bias due to intended intervention deviations	➕	➕	➕	➕	➕	➕	❔	➕	➕	➕	➖
Bias due to missing outcome data	❔	➖	➕	➕	➖	➕	➕	❔	❔	➕	➕
Bias in measurement of the outcome	➕	➕	➕	➕	➕	❔	➕	❔	➕	➕	❔
Bias in selection of the reported result	➖	➕	➕	➕	➕	➖	➕	➖	➖	➕	❔
Overall risk of bias	➕	❔	➕	➕	➕	➕	➕	❔	❔	➕	❔

Results

Our paper analyzed a total of 11 papers, and their characteristics are recorded in Table 3.

**Table 3:** Paper Characteristics AI: artificial intelligence; ECG: electrocardiogram; CNN: convolutional neural network; DNN: deep neural network; ACR: acute cellular rejection; ICM: insertable cardiac monitor; SECG: subcutaneous electrocardiogram; LSTM: long short-term memory; ML: machine learning; CHF: chronic heart failure; MI: myocardial infarction; IoT: internet of things; STEMI: ST-elevation myocardial infarction; NSTEMI: non-ST-elevation myocardial infarction; IDOVEN: Intelligent Detection of Ventricular Events Network

Author	Country	Year	Location	AI Use	Cardiovascular Condition
Martínez-Sellés and Marina-Breysse [22]	Not explicitly mentioned; general applicability suggested.	2023	Global (covers applications across clinical and real-world settings).	Interpretation and detection of arrhythmias, ST-segment changes, QT prolongation.	Arrhythmias, myocardial infarction (STEMI and NSTEMI), atrial fibrillation, ventricular tachycardia. Heart failure, drug-induced arrhythmias, long QT syndrome, and cardiovascular disease prevention.
				Risk prediction (arrhythmias, sudden cardiac death, etc.).	
				Real-time monitoring and alerting (wearables and implanted devices).	
				Signal processing (noise reduction, feature extraction).	
				Therapy guidance and optimization (e.g., drug response, device therapies).	
				Integration with imaging, genomics, biomarkers.	
Muzammil et al. [23]	Global (generalized to diverse clinical settings).	2024	Global (generalized to diverse clinical settings).	Interpretation of ECG using deep learning CNNs.	Arrhythmias, heart failure, silent cardiac illnesses, and left ventricular failure.
				Automated identification of arrhythmias, silent cardiac illnesses, and left ventricular failure.	
				Assistance in risk assessment, diagnosis, and management of cardiovascular diseases.	
				Enhanced detection in emergency department settings for timely and precise treatment.	
Salerno et al. [24]	United States (based on the affiliation of the authors with the American College of Physicians and the nature of the publication).	2003	United States (generalized to internal medicine and cardiology training settings).	The paper mentions computer-assisted ECG interpretation as an adjunct tool to support physician interpretation, though it stresses that computers are less accurate than human interpretation and should not replace trained providers.	The paper is focused on 12-lead resting ECG interpretation, a diagnostic tool for various cardiovascular conditions, but does not specify a single cardiovascular condition.
Siontis et al. [25]	United States	2021	Various healthcare settings; specific datasets and implementations are not tied to a single location.	AI-enhanced ECG analysis using CNNs.	Conditions analyzed include left ventricular dysfunction, silent atrial fibrillation, hypertrophic cardiomyopathy, and other undiagnosed systemic conditions.
Lima et al. [26]	Brazil (Study conducted primarily in the state of Minas Gerais using the CODE Study cohort).	2021	Data collected from multiple counties in Minas Gerais, Brazil, as well as external cohorts in Brazil (ELSA-Brasil) and a Chagas disease cohort (SaMi-Trop).	A DNN was developed to predict "ECG-age" based on raw 12-lead ECG tracings.	Broad focus on cardiovascular health, with specific applications in mortality prediction, left ventricular dysfunction, hypertension, low ejection fraction, coronary disease, and vascular aging.
				AI was utilized to assess cardiovascular health and predict mortality risk by comparing ECG-age with chronological age.	
Adedinsewo et al. [27]	United States	2023	Three Mayo Clinic sites	Developed a deep-learning model (AI-ECG) to detect moderate-to-severe acute cellular rejection (ACR) in heart transplant recipients using 12-lead ECG data.	Cardiac allograft rejection in heart transplant recipients
Quartieri et al. [28]	Not specified, but conducted at multiple centers (based on the authors’ affiliations).	2022	Multiple centers (exact locations not specified)	AI was used to enhance the classification accuracy of cardiac arrhythmias detected by insertable cardiac monitors (ICMs). The Willem™ AI algorithm (IDOVEN) was employed to classify subcutaneous electrocardiogram (SECG) recordings from the ICM device.	Cardiac arrhythmias (asystoles, bradycardias, ventricular tachycardias, atrial tachyarrhythmias, including atrial tachycardia and atrial fibrillation)
Dampage et al. [29]	Not specified.	2021	Not specified.	AI-based system used to monitor and classify heart sounds, with features like noise cancellation and Long Short-Term Memory (LSTM) architecture for classification.	Heart conditions, particularly heart murmurs and normal heart sounds.
Anand [30]	Not specified.	2024	Not specified.	AI-driven devices for monitoring heart health in patients with cardiac conditions, enhancing early detection and personalized care.	Heart conditions, including cardiovascular diseases and heart-related anomalies.
Krittanawong et al. [31]	Not specified.	2021	Not specified.	ML algorithms used to interpret biosensor data for near-real-time diagnosis and therapy in cardiovascular care.	Heart failure, arrhythmias, cardiovascular abnormalities, and related conditions.
Shumba et al. [32]	Italy	2023	University of Salento, Lecce, Italy; University of Salerno, Baronissi, Italy	AI and edge computing are used to process and analyze data from wearable devices in real-time, enabling personalized management of CHF. AI supports the integration of wearable devices into IoT infrastructures for patient diagnosis and management.	CHF, arrhythmias, MI, and ventricular arrhythmias.

ECG analysis AI systems

We analyzed a total of six papers on the efficacy of AI in ECG analysis, which were recorded in Table 4.

**Table 4:** AI Systems’ Efficacy in ECG Analysis AI: artificial intelligence; ECG: electrocardiogram; CNN: convolutional neural network; ACLS: advanced cardiac life support; DNN: deep neural network; ELSA-Brasil: Brazilian Longitudinal Study of Adult Health; SaMi-Trop: the Chronic Chagas Heart Disease Cohort Study; HR: hazard ratio; ACR: acute cellular rejection; AUC: area under the curve; CI: confidence interval

References	Background	Location	AI Usage	Positive Findings	Complications
Martínez-Sellés and Marina-Breysse, 2023 [22]	AI improves ECG analyses by mimicking expert cardiologists, identifying abnormalities, and integrating diverse data modalities. It has proven useful for diagnosis, risk stratification, and therapy optimization, though prospective testing is limited.	Global	N/A	High accuracy in real-time detection of myocardial infarction.	Retrospective datasets dominate current validations, lacking real-world and prospective trials.
				Enhanced patient selection for therapies like cardiac resynchronization and defibrillators.	Challenges in signal noise reduction and variability across populations.
				Improved classification of risk groups for personalized treatment.	Inconsistent cost-effectiveness analyses across health systems.
				Cost effectiveness demonstrated in select settings (e.g., hemodialysis).	
Muzammil et al., 2024 [23]	AI-enhanced ECG analysis improves diagnostic accuracy and consistency by recognizing subtle patterns undetectable to human interpreters. However, implementation is limited by biases (age, gender, race), inadequate generalization, and regulatory barriers. Addressing these issues is crucial for clinical deployment.	Global (generalized to diverse clinical settings)	Interpretation of ECG using deep learning CNNs.	Improved identification of heart failure and other conditions in emergency settings.	Systematic biases in datasets (age, gender, racial disparities).
			Automated identification of arrhythmias, silent cardiac illnesses, and left ventricular failure.	Quick and accurate detection of complex cardiac disorders.	Poor generalization across diverse populations.
			Assistance in risk assessment, diagnosis, and management of cardiovascular diseases.	Enhanced diagnostic precision and risk stratification compared to human interpretation.	Regulatory delays and validation requirements.
			Enhanced detection in emergency department settings for timely and precise treatment.	Potential for aiding clinical decision-making and improving patient outcomes.	Lack of interpretability undermines clinician trust.
					Need for diverse datasets and robust validation to ensure practical applicability.
Salerno et al., 2003 [24]	The paper addresses the need for proper training in interpreting 12-lead ECGs, as non-cardiologists often make more errors compared to cardiologists. It also discusses the potential role of computer-assisted interpretation, which can reduce errors but still requires human oversight.	United States	The paper mentions computer-assisted ECG interpretation as an adjunct tool to support physician interpretation, though it stresses that computers are less accurate than human interpretation and should not replace trained providers.	Computer-assisted ECG interpretation can help reduce errors and speed up the process but is not as accurate as physician interpretation.	Non-cardiologist physicians tend to make more errors in ECG interpretation compared to cardiologists.
				Cardiologists outperform other specialists in ECG interpretation, particularly when minimal patient history is provided.	The rate of adverse patient outcomes from ECG errors is low but still a concern.
				Residency training in internal medicine with ACLS instruction is sufficient for routine and emergency ECG interpretation.	The need for further study on the impact of patient history on ECG interpretation and additional training when the clinical history is unknown.
Siontis et al., 2021 [25]	ECGs are the most common diagnostic tools in cardiology, but human interpretation has limitations. AI, particularly CNNs, allows for deeper pattern recognition, detecting conditions that are invisible to conventional ECG analysis.	Various healthcare settings	Phenotyping cardiovascular conditions using ECG data, including identifying age, sex, race, and specific cardiac disorders.	CNNs improve diagnostic accuracy and detect conditions like silent atrial fibrillation and left ventricular dysfunction.	The "black box" nature of AI models complicates interpretability and trust.
			Integration with wearable technologies for real-time monitoring and diagnostics.	AI-processed ECGs enable previously undocumented discoveries and phenotyping opportunities.	High-quality training data and external validation are needed to ensure accuracy and generalizability.
				Potential to streamline workflow efficiency in clinical practice and enhance integration with wearable technologies.	Implementation challenges include clinician training, system integration, and ethical considerations.
Lima et al., 2021 [26]	ECGs are widely used but have limitations in manual interpretation. AI-based models offer a paradigm shift, using end-to-end DNN approaches to analyze ECG signals without relying on predefined features, enabling the detection of subtle patterns related to cardiovascular health.	Minas Gerais, Brazil + ELSA-Brasil + SaMi-Trop cohorts	Training on a dataset of 1,558,415 patients to develop an age-prediction model using ECG signals.	Patients with ECG-age >8 years greater than their chronological age showed significantly higher mortality risks (e.g., HR 1.79 in CODE-15%).	The "black box" nature of DNNs limits interpretability, making it challenging for clinicians to understand decision-making processes.
			Validation in three cohorts: (1) CODE-15% (218,169 participants). (2) ELSA-Brasil (14,236 participants). (3) SaMi-Trop (1,631 participants with Chagas disease).	Patients with ECG-age >8 years smaller than their chronological age exhibited reduced mortality risks (e.g., HR 0.78 in CODE-15%).	Reduced generalizability in external cohorts like SaMi-Trop for certain mortality risk predictions (e.g., ECG-age <8 years smaller, not statistically significant).
			Prediction of mortality risk based on discrepancies between ECG-age and chronological age.	The model performed well across diverse populations and even predicted mortality in patients with normal ECGs.	Statistical power limitations in specific subgroups (e.g., normal ECGs in ELSA-Brasil).
Adedinsewo et al., 2023 [27]	Current non-invasive methods for detecting cardiac allograft rejection have limited accuracy and integration into routine care. Severe cardiac allograft rejection has been associated with specific ECG changes, prompting the exploration of an AI-enabled ECG model to enhance detection.	Three Mayo Clinic sites	The AI-ECG model was trained and validated using 7590 unique ECG-biopsy pairs (from 1427 patients). It aimed to identify moderate-to-severe ACR using biopsy-confirmed data.	High detection performance with an AUC of 0.84 (95% CI: 0.78–0.90) in the test set.	Small sample size in the prospective study (n = 56) limits generalizability of findings.
				Sensitivity of 95% (19/20; 95% CI: 75–100%) for detecting ACR in the retrospective test set.	No discussion of potential biases or the impact of confounding variables in the abstract.
				AUC of 0.78 (95% CI: 0.61–0.96) and sensitivity of 100% (2/2; 95% CI: 16–100%) in a prospective proof-of-concept study.	

ECG serves as one of the many modalities that help in lowering the heart disease mortality rate by enabling the evaluation of the heart's conduction system [33]. Medical professionals use electrodes on patient skin to record cardiac electrical signals, which they then use to identify conduction abnormalities [34]. Electrocardiography has many common applications, including myocardial injury detection and ischemia assessment, as well as congenital heart disease and cardiac trauma, atrial fibrillation (written as Afib), and CAD diagnosis [33, 35]. The implementation of AI has enhanced ECG usage through its demonstrated accuracy in distinguishing between arrhythmic and non-arrhythmic ECG strips [22]. The integration of AI into ECG technology holds potential to fix medical errors in arrhythmia diagnosis that occur when physicians incorrectly interpret heart rhythms [36]. The AI algorithms achieve a detection accuracy of 96-97% in identifying irregular heart rhythms and left ventricular hypertrophy and outperform cardiologists in these assessments [22, 37]. AI models successfully detect patients' ventricular systolic functions in medical settings. AI predicts abnormal blood output levels through ECG-provided data [22]. The AI-ECG model demonstrated a high accuracy of 85.7% in detecting heart pumping function abnormalities through left ventricular ejection fraction (LVEF) testing. In the context of Afib diagnosis, the AI model achieved an impressive accuracy rate of almost 80%. AI-ECG showed an outstanding negative predictive value exceeding 99% for valvular heart diseases, which confirms its ability to rule out major conditions [36]. The precise diagnostic applications of AI enable healthcare professionals to make better decisions and reduce their workload [22].

Despite its diverse capabilities, ECG technology lacks the ability to detect paroxysmal rhythms, which are irregular heart rate patterns that emerge unpredictably throughout the day. A 20-minute ECG strip may not detect these irregularities [38]. Research indicates that AI demonstrates strong capabilities to forecast atrial fibrillation development from paroxysmal rhythm patterns. Researchers conducted a study to validate AI-based identification of paroxysmal atrial fibrillation from 12-lead ECG recordings obtained during different examination procedures and confirmed the models' accurate performance [22].

The research findings demonstrate that ECG AI technology has advanced to become a complex and broad artificial intelligence application used worldwide in clinical settings. These models demonstrate accuracy levels within the 80% to 90% range, which leaves room for potential errors to occur. Additional research is necessary to assess ECG technology capabilities thoroughly because more quality datasets are becoming available.

AI in continuous heart monitoring

Our analysis included five papers on AI's efficacy in continuous heart monitoring, which was recorded in Table 5.

**Table 5:** AI Systems’ Efficacy in Continuous Heart Monitoring ICM: implantable cardiac monitor; SECG: subcutaneous electrocardiogram; AI: artificial intelligence; IoT: internet of things; LSTM: long short-term memory; MFCC: mel frequency cepstral coefficient; FFT: fast Fourier transform; ML: machine learning; BP: blood pressure; HR: heart rate; PPG: photoplethysmography; AFib: atrial fibrillation; MI: myocardial infarction; VA: ventricular arrhythmia; WCD: wearable cardioverter defibrillator; CHF: congestive heart failure

References	Background	Location	AI Usage	Positive Findings	Complications
Quartieri et al., 2022 [28]	ICMs are used for long-term monitoring of patients with unexplained syncope or those at risk of cardiac arrhythmias. The large volume of data transmitted by ICMs can lead to prolonged review times to identify clinically relevant arrhythmias, necessitating the use of AI for more efficient data processing.	Multiple centers (specific locations not provided).	The Willem™ AI algorithm (IDOVEN) was applied to classify SECGs from ICM devices and compared to expert electrophysiologist classifications.	AI algorithm demonstrated 95.4% overall accuracy, with 97.19% sensitivity, 94.52% specificity, 89.74% positive predictive value, and 98.55% negative predictive value for arrhythmia classification.	The study was conducted with a relatively small sample size of 20 patients.
				AI reduced false-positive results by 98.0% overall.	The data was retrospective, which may introduce biases.
				AI significantly reduced healthcare staff workload by triaging ICM data.	Limited details on the implementation challenges or integration of AI with clinical workflows.
Dampage et al., 2021 [35]	Heart sounds provide essential physiological information, but manual monitoring and diagnosis can be time-consuming and require medical supervision. The AI-based heart monitoring system proposed aims to automate this process, enabling patients to monitor their heart sounds independently and share the data with healthcare providers via the Internet of Things (IoT).	Not specified.	The AI-based heart monitoring system utilizes LSTM for classifying heart sounds into categories like Normal, Murmur, and Artifact. MFCC is used for sound feature extraction, and FFT for signal processing.	AI system allows classification of heart sounds, enabling patient self-monitoring of heart conditions.	Details on system implementation and real-world challenges in integrating IoT and AI into healthcare are not provided.
				Use of noise cancellation and FFT improved feature extraction and system efficiency.	The system's accuracy and performance in real-world clinical settings are not clearly evaluated in the abstract.
				IoT-enabled data transfer supports remote monitoring by providers.	
				System supports personalized healthcare applications.	
Anand, 2024 [30]	The integration of AI into cardiac monitoring devices is revolutionizing patient care by improving early detection, prediction, and personalized treatment approaches for heart conditions. This review explores how AI technologies are applied in cardiac care, highlighting their potential to transform healthcare systems.	Not specified.	The paper reviews AI in wearable health tech, remote monitoring, smart devices, real-time analytics, and diagnostics. AI is used for heart rate monitoring, detecting anomalies, and predicting cardiovascular events.	AI enhances early intervention through real-time monitoring and predictive analytics.	Specific challenges in implementing AI-based devices in clinical settings are not detailed in the abstract.
				Personalized cardiac care reduces heart-related morbidities.	The long-term impact of AI-driven monitoring on healthcare practices and patient outcomes is still evolving.
				Adaptive, responsive healthcare ecosystem enabled by AI integration.	
				Continuous monitoring and personalized recommendations improve outcomes.	
Krittanawong et al., 2021 [31]	Ambulatory monitoring is crucial for cardiovascular care but faces challenges due to unpredictable events and intermittent data. ML improves diagnostic accuracy from physiological biosignals but still has hurdles in data quality, integration, and ethics.	Not specified.	ML analyzes data from wearable sensors monitoring cardiac output, BP, and rhythm. It diagnoses heart failure and arrhythmias and integrates biosignals for personalized care.	ML increases diagnostic accuracy and data actionability.	Issues remain with biosignal accuracy, interpretation, and clinical guideline integration.
				Sensors and biosignals enable continuous, personalized CV care.	Ethical, legal, and regulatory concerns (e.g., data privacy, ownership).
				Near-real-time diagnosis improves speed and reliability.	Real-world variability (e.g., noisy data, unrepresentative training sets) limits effectiveness.
				Regulatory and professional support for ML technologies is growing.	Data access and interoperability challenges hinder adoption.
Shumba et al., 2023 [32]	Smart wearables with AI and edge computing enable at-home care for chronic CV diseases like CHF, supporting real-time diagnosis and timely intervention.	Italy (University of Salento, University of Salerno)	Edge AI processes wearable biosignal data (ECG, PPG) for detecting arrhythmias (AFib, MI, VAs). AI enhances signal preprocessing and accuracy for BP and HR.	Edge AI in wearables enables CHF monitoring and early arrhythmia detection.	Some wearables may struggle with complex or noisy signals.
				Real-time analysis supports personalized care and rapid diagnosis.	Integration into clinical workflows remains challenging.
				WCDs with AI classify shockable rhythms, preventing fatal arrhythmias.	WCDs require precise tuning to avoid misclassification and inappropriate shocks.
				Multiple arrhythmias can be identified and classified automatically.	Privacy, security, and data accuracy challenges persist in clinical use.

In-hospital telemetry involves the continuous monitoring of the patient’s ECG and vitals. This type of cardiac monitoring can provide more detailed analyses to physicians due to its length. It currently assists a wide variety of patients, including those with a history of heart attacks, cases of acute coronary syndrome, and, most commonly, arrhythmias. This telemetry can also occur outside the clinical setting, allowing patients to pursue their daily activities while closely monitoring their cardiovascular system’s electrical signals. AI can further enhance these diagnoses by characterizing these waveforms in real-time and sending alerts to hospital staff when an abnormal ECG is detected, similar to a regular momentary ECG. Telemetry in clinics has become a staple and remains crucial in critical care settings [39, 40].

Similar to how AI is being used to evaluate ECG data, AI is also being utilized to design early warning systems that can analyze continuous telemetry data and predict a plethora of cardiovascular conditions. These AI programs detect and read inconsistencies in ECG strips, alerting a physician team and providing patient information, a detailed prognosis, and advice for further steps. Furthermore, AI models have removed labor-intensive monitoring processes conducted by physicians post-cardiac arrest, guiding treatment decisions [39].

Heart rhythm monitoring often also occurs outside the clinic through mobile technologies that save physicians valuable time and offer a convenient solution for patients to integrate into their daily lives seamlessly. One of these devices is the wearable remote known as the Holter monitor, which has become an increasingly common approach to determining synopses of a patient’s condition [41]. A study that demonstrated AI’s use in wearable devices enabled real-time monitoring, ensuring a sensitivity of 93% and specificity of 84% for atrial fibrillation detection [22].

The Holter monitor utilizes skin-placed electrodes for continuous monitoring. By incorporating AI into this technology, healthcare providers can ensure the longevity and efficiency of diagnoses and enhance patient care. This is evidenced by a study that tested 100 Holter electrocardiogram (ECG) recordings of 82 patients. The study found strong correlations between manual and AI-based AFib burden assessments, indicating that the AI tool is an accurate alternative for assessing AFib burden. Additionally, using this saves physicians time, especially when dealing with multiple AFib episodes [42].

Discussion

Modern AI applications enhance diagnostic accuracy and change the way we practice. Yet, each AI tool can also falter, often providing inaccurate results. More study is necessary to improve data interpretation accuracy and reliability regarding AI algorithms, especially as AI is integrated into everyday clinical practice. For example, image-based diagnostic tasks like accurate anatomical delineation in cardiac imaging and improving resolution in echocardiography are evident and flawed areas for the AI to optimize; however, improvement in AI could replace considerable time savings for physicians. Other types of AI, such as consumer wearable AI devices, happen to be particularly affected by high-mode consumption like intense exercise, which ultimately inhibits readings or makes readings worse; thus, invalidating the diagnosis (despite it being a false negative). These similar questions exist for AI that work optimally in closed or ideal testing conditions, and as they perform in patient populations, they may infrequently produce a negative result.

Throughout the integration of AI in healthcare, there is this question of AI's reliability, which has now been dubbed the "black box" problem [43]. The issue is that confusion arises from one model's answer, but there is confusion about how this model arrived at that answer, which makes it hard for medical practitioners to analyze how the AI program created their solution, or errors arise when the AI gets trained from data, based on how the data has been organized. Errors can be imported into the AI training data when there is false and incorrect information, if there are outlier datasets, and you may perceive that a result or conclusion cannot be understood [44]. In addition, if the training datasets lack demographic and clinical diversity, it is likely that those models will perform poorly on underrepresented populations, and this raises the question of bias and fairness. It is therefore important to have representative training data to improve reliability and clinical trust in AI systems. Interpretability is still a crucial challenge for clinicians, who want to be clear about how to use the output and its accountability. It is continuing to emerge within the field of explainable AI (XAI), offers solutions by focusing on creating more interpretable models. Examples of tools for XAI include SHAP (SHapley Additive exPlanations) and LIME (Local Interpretable Model-agnostic Explanations), which enable the visualization of input variable importance to the prediction of models. This contributes to a better understanding of clinical decision support by demonstrating the transparency of models. Important research processes will become imperative to embed AI into established clinical practice through the development of context-adaptive algorithms [45].

The narrative review contains multiple limitations in its methodology. The research depends on qualitative analysis of existing literature, which creates selection bias that restricts the general applicability of its results. The review lacks both meta-analysis and systematic result quantification, which would strengthen the evidence about wearable technology and edge AI effectiveness in managing chronic heart failure (CHF). The diverse research methods, study populations, and outcome measures in the included studies create difficulties for drawing uniform conclusions. The fast evolution of wearable and AI technologies makes some research findings potentially outdated or replaced by newer studies. The absence of real-world validation studies combined with the focus on theoretical or pilot frameworks restricts the practical application of these technologies in clinical settings, thus requiring additional research and clinical trials to address these gaps.

Future prospects

Overview

Several future AI applications hold great promise in cardiology, but cannot be considered applicable technologies due to their current stage of development. These tools have the potential to significantly enhance complicated scanning techniques and possibly even surgical procedures. AI is also involved in constructing sophisticated programs that assess a wide array of patients and provide specific, data-inclusive support, exceeding the quality of previously discussed data-support systems. The following sections delve into these “prototypes” in detail.

Smartwatch AI Detection

Over the past couple of decades, smartwatch AI has been designed and developed to detect and inform the user of possible heart arrhythmia [46, 47]. This innovation mitigates the predicament of expensive healthcare costs. It alleviates a physician’s schedule, allowing doctors to attend to more patients and allowing people to participate in their daily activities while keeping a check on their health. 

Modern smartwatches can detect heart rate, which can help detect arrhythmias. In the Apple Heart Study, the Apple Watch series one through three were utilized to detect any possible inconsistencies in heart rates that may indicate A-fib. Out of 419,297 participants who consented to the assessment, 2,161 (0.5%) received an alert on their smartwatch for a possible arrhythmia, of which 84% were affirmative for an irregular pulse [48]. 

The Apple Heart Study and 18 other studies indicated a smartwatch arrhythmia detection accuracy of 85-100%. Although this data appears reasonably accurate, these numbers depend on the user’s activities, as heavy aerobic exercise can hinder precision and induce errors in sensitivity [49]. Additionally, AI algorithms in most digital watch models have mostly been studied to detect A-fib, thus implying that data regarding other arrhythmias is limited [50].

Increasing research is vital to discover novel approaches to detect arrhythmias accurately in the context of intense aerobic exercise, which may aid in diagnosing exercise-induced arrhythmias. Furthermore, although smartwatches are much more convenient to wear and handle than other wearables, such as the Holter monitor, they lack accuracy and precision, decreasing their reliability in real-world applications. Finally, limited studies have explored the efficacy of smartwatch AI in detecting arrhythmias beyond atrial fibrillation (AFib), emphasizing the need for additional research to assess the effectiveness of smartwatch AI in identifying other types of arrhythmias [51].

Other AI-Powered Detection and Decision-Support Programs

Hypertension is highly prevalent when blood flow through the arteries exceeds normal pressure. Recent statistics reveal that around half of all Americans develop high blood pressure, with cases varying in severity and worsening with age. When characterizing blood pressure in a numerical form, there are two numbers: systolic blood pressure (also referred to as SBP, which occurs when the ventricles pump blood out of the heart to the body) and diastolic blood pressure (also referred to as DBP, which occurs when blood travels into the heart) [52, 53]. To approach this current predicament, Boston University’s novel medication support model has proven effective in prescribing the optimal treatment of hypertension based on the patient’s medical history, demographics, and medical test results. Real-time advice, including the probability of success for each treatment therapy, is then given to physicians, aiming to revolutionize hypertension treatment at the individual level [54, 55].

This all-inclusive model’s efficacy was authenticated in a study conducted by researchers affiliated with Boston University [56]. The study's dataset included 42,752 patients with hypertension. By employing a prescriptive policy design, the model considered one out of seven types of antihypertensive drugs. The model’s performance was compared with other algorithms, exceeding other proposed models significantly and performing 7.08% better than the second-best model (50). BU’s model primarily emphasizes mitigating SBP levels (possibly due to its strong correlation with the development and significance of the metric in hypertension management versus DBP levels), evidenced by its SBP reduction of 14.22 mmHg, 70.3% more effective than modern healthcare [56]. The model's recommendation policy provides physicians with a list of recommended agents and their associated probabilities of success. It demonstrates potential benefits in deprescribing (withdrawing medication to a certain amount to improve patient outcomes), showing improved SBP reduction in patients on multiple medications [57]. The study emphasizes the model's potential to modify treatment for better health management. Furthermore, the program’s transparency is also noted to help improve physicians’ trust in AI-developed solutions, possibly helping to persuade more people to welcome AI’s development without issue [58-60].

Another decision support tool, the ASSIST (Anatomical vs. Stress testing Decision Support Tool), has emerged as a groundbreaking algorithm in cardiology. It uses sophisticated machine-learning techniques to revolutionize decision-making for patients with chest pain [61]. Developed from data collected from the PROMISE (PROspective Multicenter Imaging Study for Evaluation of Chest Pain) trial involving 9,572 patients, the tool utilizes a spatial data representation created through Cox regression models (a statistical method that models the survival time for patients) and gradient boosting algorithms (another machine learning technique that builds a predictive model typically in the form of decision trees where each new tree corrects the errors made by the previous one, creating an iterative process) [62, 63]. ASSIST operates by examining 57 different factors before individuals are randomly assigned to specific groups. It assesses each group member’s risk of major adverse cardiovascular events (MACE). Using data from the PROMISE study, it predicts potential outcomes based on a specific heart test. ASSIST demonstrated its effectiveness when tested on the remaining 20% of PROMISE participants and another group from the SCOT-HEART trial. It is notably reducing overall mortality and heart attacks. This tool provides another example of how machine learning can offer personalized guidance for managing heart conditions based on each individual's unique details [61]. 

This all-inclusive model’s efficacy was authenticated in a study conducted by researchers affiliated with Boston University [56]. The study's dataset included 42,752 patients with hypertension. By employing a prescriptive policy design, the model considered one out of seven types of antihypertensive drugs. The model’s performance was compared with other algorithms, exceeding other proposed models significantly and performing 7.08% better than the second-best model [50]. BU’s model primarily emphasizes mitigating SBP levels (possibly due to its strong correlation with the development and significance of the metric in hypertension management versus DBP levels), evidenced by its SBP reduction of 14.22 mmHg, 70.3% more effective than modern healthcare [56]. The model's recommendation policy provides physicians with a list of recommended agents and their associated probabilities of success. It demonstrates potential benefits in deprescribing (withdrawing medication to a certain amount to improve patient outcomes), showing improved SBP reduction in patients on multiple medications [57]. The study emphasizes the model's potential to modify treatment for better health management. Furthermore, the program’s transparency is also noted to help improve physicians’ trust in AI-developed solutions, possibly helping to persuade more people to welcome AI’s development without issue [58, 64].

## Conclusions

This narrative review demonstrates how wearable technologies and edge AI systems can transform the management and prevention of CHF. The combination of real-time monitoring and early arrhythmia detection, and personalized interventions through these technologies leads to better patient results and decreased healthcare expenses. The clinical adoption of these technologies faces obstacles because of privacy concerns and device connection issues, and the requirement for extensive real-world testing. The complete realization of these innovations depends on addressing their current limitations through additional research and development efforts. Wearable and AI-driven technologies show promise to transform CHF management by providing patients and healthcare providers with advanced care delivery tools.
